# Sustain release of dexamethasone from polyvinyl alcohol microparticle produced via coaxial microfluidic system

**DOI:** 10.1186/s13104-023-06544-3

**Published:** 2023-10-12

**Authors:** Melika Abdi Majareh, Seyed Mohammad Davachi, Yasaman Tavakoli Moghaddam, Mehdi Khanmohammadi

**Affiliations:** 1 Department of Microbiology, Karaj Branch, Islamic Azad University, Karaj, Iran; 2 Department of Biology and Chemistry, Texas A&M International University, 78041 Laredo, TX USA; 3 Skull Base Research Center, The Five Senses Institute, School of Medicine, Iran University of Medical Sciences (IUMS), Tehran, Iran; 4 Biomaterials Group, Materials Design Division, Faculty of Materials Science and Engineering, Warsaw University of Technology, Warsaw, Poland

**Keywords:** Polyvinyl alcohol derivative, Microparticle, Microfluidic technique, Dexamethasone, Controlled release.

## Abstract

**Objective:**

Polyvinyl alcohol (PVA) as a synthetic biopolymer has unique physicochemical properties to achieve an efficient drug carrier. In this study phenol-substituted polyvinyl alcohol (PVAPh) microparticle was made through a microfluidic system and peroxidase-mediated reaction in the presence of hydrogen peroxide and in following dexamethasone (Dex) release characteristics from this vehicle were elaborated for sustained drug delivery applications.

**Results:**

PVAPh was synthesized by esterification and amidation reactions respectively. Then, the synthesized PVAPh solution containing peroxidase and Dex flowed within the inner channel of the coaxial microfluidic device while liquid paraffin saturated with H_2_O_2_ flowed from the outer channel. The monodisperse microparticles were produced in a spherical shape with an average diameter of 160 μm. The Dex was successfully encapsulated in PVAPh MP and its sustained release profile was maintained for up to 7 days. It was found that exposure of Dex-loaded PVAPh MPs to subcultured mouse embryonic fibroblast 10T1/2 cells had no deleterious effects on cell viability, morphology and growth rate. Moreover, the sustained release of Dex and the high mechanical durability of PVAPh MPs suggest an excellent prospect for the synthesized PVAPh and the developed method as a biocompatible carrier required for drug delivery and regenerative medicine.

## Introduction

Polyvinyl alcohol (PVA) is a well-known hydrophilic and degradable polymer which has been widely utilized in biomedical applications [1–3]. It has attracted as an excipient scaffold and drug/cell carriers in pharmaceutical sciences and pharmaceutical industry due to its tissue adhesiveness, amendable mechanical properties, ease processability and high biocompatibility resulting in weak antigenic impacts [3–5]. The PVA utilized in tissue engineering is mainly modified and crosslinked to form three-dimensional (3D) constructs through many ways of chemical crosslinking methods [1, 6]. Among them, horseradish peroxidase reaction is bonded activating phenolic groups on backbone of PVA by carbon to carbon and oxygen coupling [3, 4]. The nucleophile in the HRP reaction is hydrogen peroxide which attacks the heme ferric ion [7, 8]. The major advantage of HRP compared with other enzymes reported to be effective for obtaining hydrogels, such as transglutaminase and tyrosinase, is more rapid hydrogelation which is essential precursor fabrication of microparticles through microfluidic technology [3, 7, 9]. Recently, the PVA is modified by introducing phenol (Ph) moieties to provide required crosslinkable groups in HRP/H_2_O_2_-mediated reaction [3, 4, 10]. The desired manageable properties of hydrogel vehicles obtained from this enzymatic reaction and microfluidic system possibly pave the way for using PVA features to produce biocompatible microparticle that is an attractive structure for different usages in biomedical fields [9–12]. Besides, dexamethasone, a potent synthetic glucocorticoid, plays a crucial role in the control and management of numbers of diseases including chronic rhinosinusitis, which is inflammation and infection of the nasal cavity and paranasal sinuses that lasts longer than 12 weeks [13, 14]. Nonetheless, the likelihood of its systemic side effects, such as weight gain, immune suppression, and adrenal suppression, underscores the importance of employing controlled delivery systems for sustained and localized drug administration [15, 16].

**Fig. 1:**
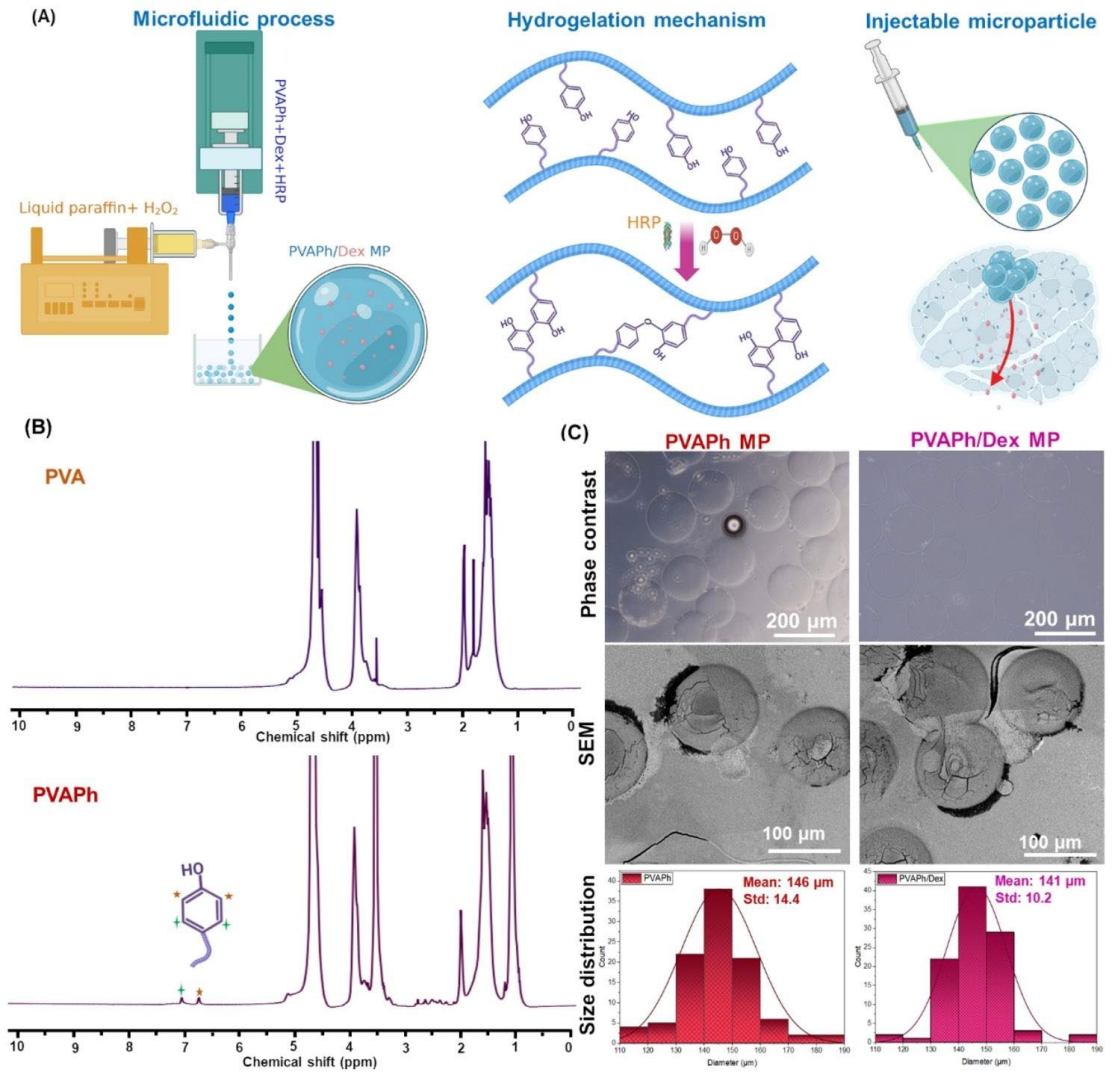
**(A)** Schematics of PVAPh microparticle fabrication, hydrogelation and its injectability. **(B)**^1^ H-NMR spectrums of intact PVA and synthesized PVAPh. **(C)** Microphotograph, SEM and size distribution of fabricated PVAPh microparticles containing Dex

In this study, we used the same technique to examine the possibility of PVAPh microparticle production and encapsulation of Dex within such vehicle for sustained drug delivery applications. The PVAPh microdroplets were formed through water in oil emulsion process of the aqueous PVAPh precursor in the oil phase, which is well-controlled by a coaxial microfluidic flow and concentration adjustment (Fig. 1). In the meantime, the HRP catalyzed the transfer of electrons from H_2_O_2_ supplied by the oil phase and subsequently PVAPh microparticles became polymerized through conjugation of Ph groups. The physical and biochemical properties of Dex-loaded PVAPh microparticles including mechanical properties, swelling rate, Dex release and cellular viability were elaborated and could provide confidential results for the applicability of PVA-based microparticles for drug delivery and regenerative medicine.

## Main text

## Materials and methods

Poly vinyl alcohol (PVA; Mw 9,000–10,000, 80% hydrolyzed), dexamethasone, HRP (200–250 units/mg), n-hydroxysuccinimide (NHS), 2-morpholinoethane sulfonic acid (MES), tyramine hydrochloride, water-soluble carbodiimide hydrochloride (WSCD), 4-dimethyl aminopyridine (DMAP), succinic anhydride (SA), dexamethasone sodium phosphate and ethanol were purchased from Sigma (Saint Louis, MO, USA). Cell proliferation reagent (WST-1) was obtained from Merck (Darmstadt, Germany).

### Synthesis of PVAPh and microparticle production

Chemical modification of PVA was carried out according to previous reports by mixing PVA with SA and DMAP for preliminary carboxylation and in following step with harvested PVA substrate with tyramine, NHS and WSCD for phenolation [3, 4]. The successful Ph conjugation was measured using the UV absorption of tyramine at 275 nm and H-NMR. The PVAPh microparticles containing Dex were prepared using lab-made coaxial double orifice microfluidic device [9, 17]. Briefly, 120 mg PVAPh was dissolved in 2 mL PBS solution at 70 °C then was allowed to reach room temperature. Then, 2 mg Dex and 300 units HRP were added and the mixed solution was poured in plastic syringe connected in syringe pump. Liquid paraffin (60 mL) containing 4% (w/v) lecithin was saturated with 150 µL H_2_O_2_ using homogenizer and poured in plastic syringe which connected to another syringe pump. The prepared polymeric solution (50 µL/min) and liquid paraffin (1.5 mL/min) were flowed into coaxial double orifice device as internal and external fluids respectively. The microparticles were formed and then collected into corning tube. After 10 min standing for diffusion of H_2_O_2_ from liquid paraffin into droplets and completion of crosslinking. The microparticles were recollected through filtration using sieving device (100 μm cell strainer) and centrifugation to harvest them without any oil residues. The average diameter of the produced PVA-based microparticles was determined using microphotographs of samples. The microparticles were freeze-dried, and stored in low temperature at -20 ^o^C. The average size, size distribution and surface morphology of microparticle were elaborated using inverted light micrograph and scanning electron microscopy.

### Physical properties of PVAPh microparticles

To evaluate the durability and stability of produced microparticles under stress conditions, specific numbers of microparticles were purred into an eppendorf tube containing 1 mL PBS and shaken using a rotary stirrer at 100 rpm and 90 ° degree position. In each time-lapses, the intact microparticles were collected through centrifugation and counted microscopically. The percentage of intact microparticles was quantified by the number of undamaged microparticles versus the number of initial microparticles [9, 11]. Besides, to realize water absorption capacity of microparticles, a quantity of the dried PVA-based microparticles (≈ 10 mg) was soaked in 10 mL PBS and kept at 37 °C until 24 h. In different time intervals, the PVAPh micoparticles were collected by pouring on paper filter and removing excess water and the swollen samples were weighed (W_1_). During isolation of the excess PBS from the swollen microparticles, unwanted loss of small amount of the used microparticles may occur. So, the swollen samples were dried at 55 °C for 8 h and their weight were measured (W_2_). Swelling degree of particles were quantified using below equation.

Swelling ratio = [(W_1_- W_2_)/ W_2_] × 100.

### Encapsulation efficacy and release profile of dexamethasone

The percentage of Dex loading and encapsulation efficiency were measured based on the following formula according previously report [15, 18]. The quantification of Dex values was performed by ultraviolet-visible (UV-Vis) spectrophotometry at a wavenumber of 242 nm (n = 3). Also, percentage of accumulative Dex release was measured by using a calibration curve. For this, 200 µL PVAPh/ Dex microparticles were suspended in 10 mL PBS buffer shaking incubator at 50 rpm and 37 °C. The aliquot sample was exchanged by fresh PBS at different time intervals. Then, the Dex content of sample solutions were measured by a UV spectrophotometer. Also, the Dex release kinetic from microparticles was examined via linear fitting of three kinetic models: zero-order, first-order and higuchi [9, 19]. Finally, each graph of the model was plotted, and the R^2^ value determined the most appropriate fitted model (Table 1).

**Table 1:** Kinetic model equations for released Dex from PVAPh microparticles

Model	Equation	Parameters	Graph
Zero-order	Q_t_ = Q_0_ + K_0_t	Q_t_: Amount of Dex released at time t Q_0_: Initial amount of Dex in the solution K_0_: Zero-order release constant	Cumulative percentage of Dex release vs. Time
First-order	Log C = Log C_0_ − K_1_t/2.303	C: Percent of drug remaining at time t C_0_: Initial concentration of the Dex K_1_: First-order release constant expressed in time^− 1^	Log percentage of Dex remaining vs. Time
Higuchi	Q = K_H_ t^1/2^	Q: Cumulative amount of Dex released in time t K_H_: Higuchi dissolution constant	Cumulative percentage Dex release vs. square root of time

### Cellular viability

To evaluate the performance of the PVAPh/ Dex hydrogel microparticle as Dex carrier, a mitochondrial activity measurement as an indicator of cell proliferation was performed by using WST-1 assay kit [20]. The mouse fibroblast 10T1/2 cells, as a model cell line, at 2.5 × 10^4^ cells/mL were seeded into a 24-well plate at 1 mL/well for 8 h. Next, the 200 µL PVAPh microparticle samples were poured into each well. At specific time points, WST1 assay was used to measure amount of water-soluble formazan dye derived from a tetrazolium salt.

## Results

### Characterization of PVAPh and microparticle

The appeared new peaks for PVA-COOH at about 2.6 ppm attributed to methylene protons of succinic acid and subsequently, the new peaks from 6.8 to 7.4 ppm, referring to aromatic protons of conjugated Ph groups at ortho and meta positions (Fig. 1B) [3, 4]. The UV spectroscopy result indicated that the amount of conjugated Ph moieties on PVAPh was 1.95 × 10^− 4^ mol-Ph /g-PVAPh. Figure 1C shows the morphology and size of the PVAPh MPs obtained via the microfluidic technique. An average size of PVAPh MPs were about 165 μm and had poisson-like distribution which proved the designed system could be finely control the size of PVAPh MPs through selecting suitable flow velocities in microfluidic channels. As the results showed both of the PVAPh MPs and even PVAPh/ Dex MPs possessed narrow size distribution and drug encapsulation did not change their structural uniformity and size distribution (Fig. 1C).

### Physical characteristics of PVA microparticle

**Fig. 2:**
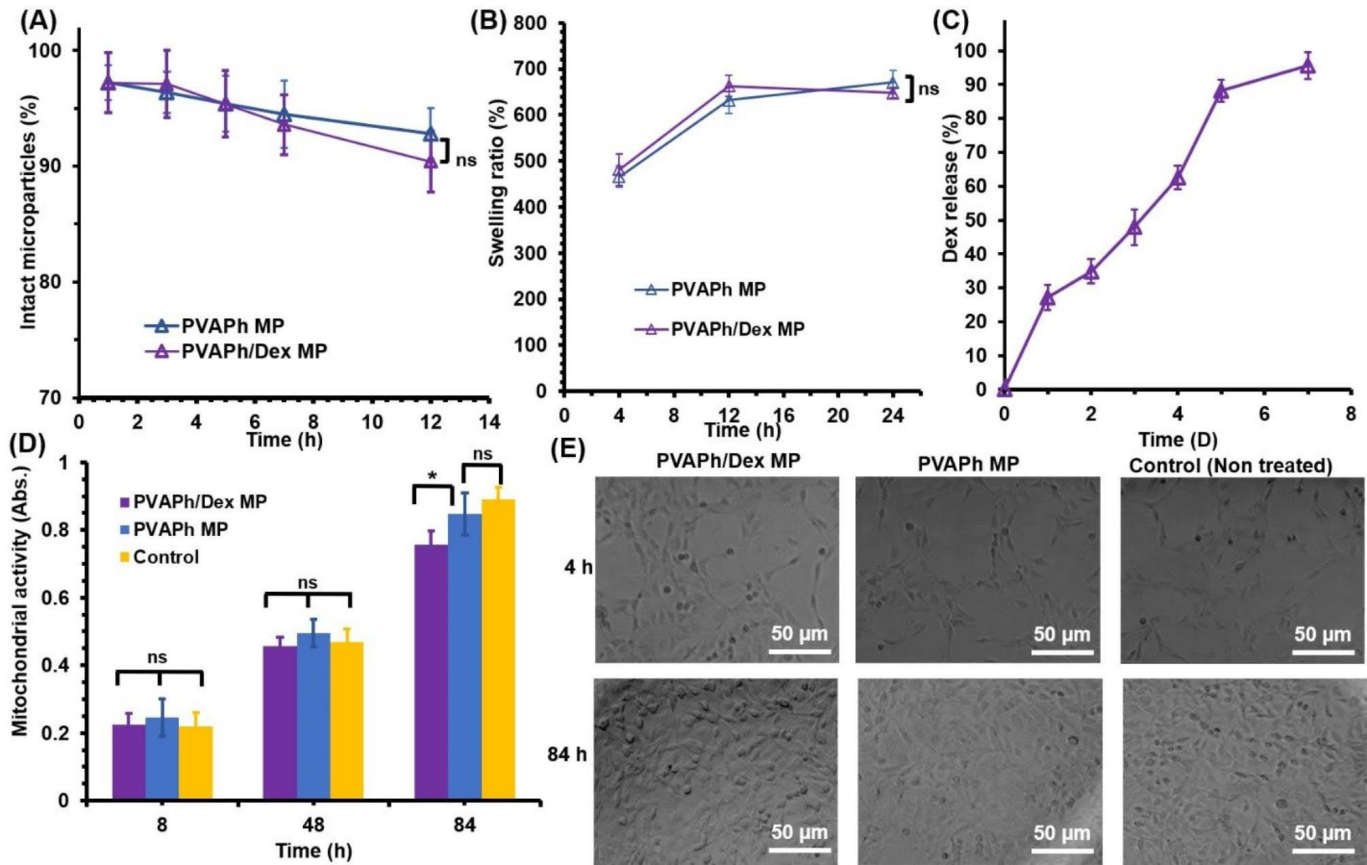
**(A)** PVA-based microparticle resistance in exposure of dynamic fluid stresses. **(B)** Swelling behaviour of microparticles at 37 °C and pH = 7.2. **(C) **Release profile of dexamethasone. **(D and E)** Mitochondrial activity **(D)** and microphotographs **(E)** of sub-cultured 10T1/2 cells in exposure of microparticles

Here, the durability of MPs was tested by subjecting them to dynamic shear flow (Fig. 2A). The significant amount of microparticles in exposed stress remained stable and intact (> 90%) in both conditions until 7 days and there were not significant differences between PVAPh MPs samples with and without Dex which means the hydrogel network did not change by encapsulation of Dex substrate. A swelling test was performed to investigate the water absorption capacity of the PVAPh MPs (Fig. 2B). A significant water uptake was observed for both of the Dex loaded and free PVAPh MPs in initial hours of test in close values for PVAPh (486% ±15) and PVAPh/ Dex (468% ± 14). The swelling ratio reached to equilibrium condition at 12 h of incubation at about 610% for both samples which means repeating unit in the PVAPh macromer and covalent bonds of Ph groups affected the swelling ratio of the PVAPh MPs and presence of Dex molecules did not insert specific negative impacts on swelling ratio of microparticles.

### Dexamethasone release analysis

The loading capacity and encapsulating efficiency determined 1.2 ± 0.2% and 68 ± 6% respectively. The Dex release kinetics from PVAPh MPs was evaluated to realize the potential of prepared microparticles (Fig. 2C). The Dex was released from the PVAPh MPs up to 28% within the initial first day which attributed to active drug on surface of microparticle. However, the microparticles showed a milder release until 7 days which could be interpreted with bulk erosion pathway of hydrogel structure as well as hydrogel dissociation during this period (Fig. 2C**)**. The regression coefficient values (R^2^) indicate appropriate kinetic model for Dex release and the proper fitting equation as the value be closer to 1 (Table 2) [19, 21]. The Dex release kinetic from PVAPh microparticle exhibited the fittest rate with higuchi model due to the higher regression coefficient to 1 [19, 21, 22]. The zero-order and first-order showed lower fitness and values of regression coefficient (Table 2).

**Table 2:** Regression coefficient of kinetic models fitted for releasing Dex from PVAPh microparticle

Samples	Zero-order		First-order		Higuchi	
	K_0_ (µg. mL^− 1^. h^− 1^)	R^2^ (-)	K_1_ (h^− 1^)	R^2^ (-)	K_H_ (µg. mL^− 1^·h^− 1/2^)	R^2^ (-)
	0.062	0.846	0.0051	0.925	1.256	0.962

### Cytocompatibility of PVA based microparticles

The cell viability was more than 93% for all experimental conditions and there was not specific difference among them on days 1 and 4 (Fig. 2D **and E**). Also, the treated 10T1/2 cells with microparticles proliferated well and the rate of mitochondrial activity demonstrated about 3 times rise of cellular activities in days 4 of incubation which means that the prepared PVA-based MPs as well as encapsulated Dex did not have adverse effects on cellular growth and morphologies.

## Discussion

The aim of this study was development of PVA-based MPs through enzyme mediated crosslinking reaction and microfluidic system and in following, to demonstrate its applicability for encapsulation of Dex within this vehicle for drug delivery purposes. The chemical characterization tests showed striking differences between PVA and PVAPh peaks which verified successful synthesis progression by two sequential esterification and amidation reactions [2–4]. The amount of conjugated Ph moieties on PVAPh was high enough for production of microparticle via microfluidic system and enzyme-mediated crosslinking likewise MP production from other polymers modified with Ph moieties [11, 17, 23, 24]. The production of PVAPh MPs proceeded by utilizing coaxial double orifice microfluidic device in water-oil emulsion system. The PVAPh droplets extruded from extruding channel was collected and hydrogel MPs obtained through proceeding HRP-mediated crosslinking reaction. We did not observe any structural instability, including coalescence of PVAPh hydrogel MPs, which means proper concentration of PVAPh solution, reactants and volumetric flow of solutions in utilized flow focusing microfluidic device. In addition, the production of biological carriers in diffusible limit size for cellular construct (200 μm<) as well as homogeneity in size distribution which are achieved here are important factors in biological compound encapsulation studies since encapsulated cells could receive sufficient nutrients and oxygen through diffusion mechanism and also each vehicle has equal quantity of bioactive compound [9, 17, 24]. These properties give us this opportunity to utilize PVA-based MPs for cell or drug delivery in determined concentration of cells or biomolecule respectively. Besides, the fabricated PVAPh MPs could be used as an injectable sample for in vivo studies without specific concerns for sample transition through the injecting needle channel and also in minimum invasiveness [25, 26]. The stability of the PVAPh MPs under exposure of dynamic fluid was significantly high which credit applicability of these vehicles in vivo or perfusion cell or tissue culture models in vitro which has dynamic fluid media [27, 28]. The MP capability as a localized drug depot is important factor for its usage in drug delivery and regenerative medicine [15, 25]. The main quantity of encapsulated Dex were released during the initial few days which could be due to high surface area of PVAPh Mps and shorter route for diffusion to their external surrounding environment [21, 22]. To precisely realize governing release mechanism of Dex form produced MPs, we evaluated the Dex release with three known models in drug delivery systems. The first-order regression coefficient means poorly water-soluble drug encapsulated in a water-soluble microparticle matrix and zero-order model’s regression coefficient related to the low solubility of Dex in the release environment **(**Table 2). The Higuchi model showed fit well for release profile of Dex which is consistent for low concentration of active agent in the matrix, where the solubility and release happen through the porosity of the hydrogel matrix [21, 22, 25]. Interestingly, the PVAPh MPs were cytocompatible and cells grew properly which indicates potential applicability of prepared PVAPh MP as a biological vehicle.

### Limitation

The obtained outcomes have ensured a commendable level of shape fidelity, structural integrity, strength, and resistance for PVA-based vehicles, intended for applications in drug delivery. However, Dex encapsulation within PVAPh microparticles exhibited rapid release kinetics over a span of a few days. To attain sustainable drug release objectives, it is imperative to enhance the control over encapsulation efficiency for extended durations. Furthermore, the size of the proposed microparticles presents a challenging aspect, affecting the achievement of optimal drug delivery efficacy. It is expected that by considering additional crosslinking of PVAPh as well as surface modification of PVAPh or suspending microparticles in other injectable bulk hydrogel could promote release time and drug delivery efficacy.

Also, the prepared hydrogel microparticles have to immediately after fabrication process freeze dried since drug continuously would release in wet condition. Besides, there is some challenge for collecting microparticles for physical evaluation of microparticles which is needed to precisely normalize data for lost quantity of samples during the experiment. It is recommended after optimization of hydrogel condition for drug delivery, examine the developed microparticles for in vivo.

## Conclusion

Monodisperse PVAPh microparticle produced by using microfluidic flow focusing and processing of covalent bonds among Ph residues. The microphotographs and statistical analysis showed the uniformity in size of microparticles as a proof of concept for the applicability of microfluidic system for PVAPh microcarrier production and encapsulation of Dex molecules. The biophysical and biochemical properties of the MPs showed that the prepared MPs could withstand dynamic fluid stress for a long time, and seeded cells in presences of Dex-loaded PVAPh microparticles remained viable and proliferated well. Therefore, the fabricated PVAPh microparticles have advantages for many biomedical applications, including drug delivery and regenerative medicine.

## Data Availability

The data created and analyzed during the current study are available from the corresponding author upon reasonable requests.

## Abbreviations

PVA: Polyvinyl alcohol
Dex: Dexamethasone
HRP: Horseradish peroxidase
H_2_O_2_: Hydrogen peroxide
DMAP: 4-dimethyl aminopyridine
SA: Succinic anhydride
WSCD: water-soluble carbodiimide hydrochloride
NHS: N-hydroxysuccinimide
MES: 2-Morpholinoethanesulfonic acid, monohydrate
NMR: Nuclear magnetic resonance
Ph: Phenol
UV–Vis: Ultraviolet–visible spectroscopy
